# CD9: Differential expression of normal bone marrow cellular components and leukemic myeloid blasts

**DOI:** 10.1093/ajcp/aqaf087

**Published:** 2025-09-17

**Authors:** Afreen Jasim, Winston Lee, Huiyan Ma, Elizabeth Quirk, Joo Song, Scott Hwee, Jessica Hughes, Parastou Tizro, Lori Soma

**Affiliations:** Department of Pathology, City of Hope Medical Center, Durate, CA, United States; Department of Pathology, City of Hope Medical Center, Durate, CA, United States; Department of Computation and Quantitative Medicine, Beckman Research Institute, City of Hope Medical Center, Duarte CA, United States; Department of Pathology, City of Hope Medical Center, Durate, CA, United States; Department of Pathology, City of Hope Medical Center, Durate, CA, United States; Department of Pathology, City of Hope Medical Center, Durate, CA, United States; Department of Pathology, City of Hope Medical Center, Durate, CA, United States; Department of Pathology, City of Hope Medical Center, Durate, CA, United States; Department of Pathology, City of Hope Medical Center, Durate, CA, United States

**Keywords:** flow cytometry, acute myeloid leukemia, CD9, normal myeloid blasts, hematopoietic stem cells

## Abstract

**Objective:**

Research on CD9 expression has been extensive in B lymphoblastic leukemia, with fewer studies focusing on acute myeloid leukemia (AML). We investigated the usefulness of CD9 in differentiating normal from abnormal myeloid progenitors, as well as expression in normal cell types and in AML.

**Methods:**

Flow cytometry was used to assess the level of CD9 expression on normal and leukemic myeloid blasts and other normal bone marrow populations. Geometric mean fluorescence intensity levels and expression patterns were compared among cell types and AML subtypes.

**Results:**

In normal subsets (n = 69), the level of CD9 expression was lowest in mature B cells, myeloid blasts, promyelocytes, and neutrophils, with intermediate expression in monocytes and highest in hematogones (stages 1 and 2). Committed myeloid progenitors (CMPs) had lower expression than hematopoietic stem cells (HSCs). CD9 typically has higher expression in AML (n = 58) compared to normal myeloid blasts and promyelocytes, and it is differentially expressed in AML, with the highest expression in *PML::RARA* AML.

**Conclusions:**

Aberrant CD9 expression can be useful differentiating normal from abnormal myeloid progenitors, with the highest level of expression in AML with *PML::RARA* in our cohort. There was differential expression between HSCs and CMPs in the small numbers studied. Normal mature B cells can be used as an internal negative control in most cases.

**KEY POINTS**

- CD9 is often higher in leukemic myeloid blasts compared to normal myeloid blasts and appears differentially expressed on normal hematopoietic stem cells vs committed myeloid progenitors.
- Normal mature B cells can be used in most cases as an internal negative control.
- CD9 is differentially expressed in acute myeloid leukemia subtypes, with acute promyelocytic leukemia having the highest level of expression.

## INTRODUCTION

CD9, first identified by Kersey et al^1^ as a cell surface antigen in leukemic lymphoblasts, belongs to the tetraspanin family. Depending on the associated proteins and cell types, the cellular function of CD9 is broad, ranging from cell adhesion and migration to proliferation, cell growth, apoptosis, and differentiation.^2^ CD9 knockout mice display no overt abnormalities, except for reduced fertility in females secondary to deficiencies in sperm-egg fusion.^3^ In human cancers, CD9 has been implicated in the promotion of various carcinogenic processes such as angiogenesis, tumor engraftment, invasion, and metastasis.^4^ Given that the association of CD9 with cell and protein type determines functionality, there is variation in prognosis related to its expression in hematopoietic and nonhematopoietic neoplasms.

In hematologic malignancies, it has been extensively studied in B lymphoblastic leukemia (BLL), associated with unfavorable outcomes and disease progression.^5-8^ Its expression in acute myeloid leukemia (AML) has also been studied, although not to the degree of BLL. Touzet et al^9^ observed that CD9 expression was increased in 40% of AML cases and was associated with a favorable prognosis.^9^ Other studies explored CD9 as a useful marker in identifying acute promyelocytic leukemia (APL), including as a marker of residual disease,^10-14^ and AML with the *NPM1* mutation in certain cohorts.^15^ In contrast, AML with *RUNX1::RUNX1T1* was reported to have lower CD9 expression based on publicly available gene expression data.^16-18^

In normal subsets, CD9 expression has been reported on a variety of hematopoietic cells, such as megakaryocytes, mast cells, basophils, eosinophils, immature B cells, and lymphocytes, at various stages of maturation and activation.^2^ CD9 is also thought to link with FC receptors on macrophages, having a role in the inflammatory response,^2,19^ with higher expression on CD14-positive, CD16-negative monocytes.^20^ There are a few studies evaluating CD9 on mature B cells, primarily assessing human peripheral blood B cells, reporting CD9 as minimal or showing small subset expression.^2,21,22^ Similarly, there are a few studies assessing CD9 on neutrophils and normal myeloid blasts, reporting absent and low to absent expression, respectively.^9,23,24^ Additionally, some investigators report differential expression between normal myeloid blasts and the hematopoietic stem cell component (HSC). Clay et al^23^ report that CD9 is variably expressed on CD34-positive cells and more prominently expressed (CD9 mid as opposed to low) in the “primitive progenitors” that are CD38 low, CD34 high.^23^ They noted the highest level of CD9 on B lymphoid and megakaryocytic committed progenitors. Alternatively, others report very low/barely detectable levels of CD9 on normal HSCs (CD34 positive and CD38 negative), with significantly higher expression on lineage-committed CD34- and CD38-positive progenitors; however, it is not clear that the CD34/CD38-positive committed progenitors were separated into myeloid and B lineage.^25,26^

Through assessment of routine clinical data in our laboratory, we had appreciated that CD9 was often expressed at higher levels in leukemic myeloid blasts compared to normal myeloid blasts and sought to explore its usefulness in determining aberrancy for potential future application as a measurable residual disease (MRD) marker.^14,24^ Given the reported historic data, we intended to assess more numerous normal cases to evaluate for potential variability of CD9 expression in normal myeloid blasts and to confirm which normal cell type would be the best internal negative control. We compared CD9 levels by multiparametric flow cytometry (MFC) between normal bone marrow cell types and leukemic myeloid blasts to confirm differential expression and which normal internal control(s) could be used. Additionally, we compared the levels of CD9 expression in leukemic blasts, using the most updated classification schemes, comparing subtypes not included in previous studies (APL often omitted when comparing large AML cohorts).^9,15^ Given that this study is retrospective, utilizing the clinically available MFC assay performed in our clinical laboratory for patients with newly diagnosed acute leukemia, we were limited to reviewing the panels already performed for our patients with AML.

## MATERIALS AND METHODS

This single-center study was approved by the institutional internal review board (IRB# 14225) and was not grant funded. The MFC samples that included CD9 analysis performed as part of clinical care, collected from October 2022 to November 2023, were obtained from the Department of Pathology archive. October 2022 was the starting date, as that was the time point that CD9 was incorporated into the leukemia/lymphoma flow cytometry panel.

### Normal cell subsets cohort

Nonmyeloid neoplasm samples (predominantly bone marrow with a few peripheral blood [PB] samples) included staging marrows from patients with lymphoma (non-Hodgkin and Hodgkin), posttherapy marrows (mature B-cell and T-cell neoplasms, BLL, plasma cell neoplasms), and marrows from patients without neoplasia (sickle cell anemia). Peripheral blood from 2 patients with solid tumors who had received granulocyte colony-stimulating factor (G-CSF) and PB from 2 patients with a history of BLL were also analyzed. None of the patients had a history of a myeloid neoplasm or current marrow myeloid abnormalities (clinically, morphologically, or cytogenetically); however, some were continuing to receive therapy for their neoplasm. Cases were sequentially obtained from the Department of Pathology archives that were adequately cellular for routine analysis, including confident separation of the specific populations. Cases that could not be separated confidently or did not have a significant population size (at least 50 events) to assess CD9 were not included. The electronic medical record (EMR) was reviewed to determine if the patients received G-CSF within 7 days prior to their flow study. Additional cases where patients had received G-CSF were sought after the original accrual to increase numbers in that cohort. Fluorescence minus 1 (FMO) data were collected at the time of analysis for myeloid blasts and mature B cells in a subset of cases. The HSC subset (CD34 positive/CD38 decreased to absent/CD19 negative) was also compared to the committed myeloid progenitors (CMPs; CD34 positive/CD38 positive/CD19 negative) in cases where the HSC component was more than 30% of the CD34+/CD19– progenitors.

### AML cohort

Original diagnostic AML cases (bone marrow) were sequentially, retrospectively collected from the Department of Pathology archives and evaluated from October 2022 to November 2023. The EMR was reviewed to confirm that only cases that were newly diagnosed as AML (including those with prior myelodysplasia that had progressed) were included. Patients with a prior diagnosis of AML or who had received prior cytoreduction therapy or prior therapy for AML were excluded. Both the International Consensus Classification (ICC) and World Health Organization fifth edition classifications^27,28^ were used concurrently to subcategorize AML, where similar, and the ICC classification for AML with the *TP53* mutation, where applicable. Patients who additionally had MFC performed on blood prior to therapy were also identified from the EMR to compare the geometric mean fluorescence intensity (GMFI) between blood and marrow. The EMR was reviewed to identify patients who were being monitored for residual disease and to assess CD9 posttherapy. Adequacy for inclusion for the follow-up/MRD cohort required an adequate cellular sample to include testing for routine MFC that included CD9 (100 000 events collected), MRD MFC testing (1 000 000 events collected), lack of components that could potentially confound the interpretation (eg, basophils in a CD34-negative AML), cytogenetic and/or molecular testing where applicable, and a low blast population (<5% of cellularity) that could still be detected by routine MFC (given only 100 000 events were collected in the assay with CD9).

### MFC methods

Each sample was bulk lysed twice using 10% BD PharmLyse Lysing Buffer (BD Biosciences), followed by a wash step using BD Pharmingen Stain Buffer (BD Biosciences). Cell density was determined using the Cellometer Auto T4 instrument, and the samples were adjusted to a target concentration of 5 × 10^6^ per milliliter. Each sample was then stained and incubated at 2 °C to 8 °C for 20 minutes with the appropriate panel. The “acute leukemia panel” was used in the diagnostic AML cases, and the Pre-B tube from that panel was additionally assessed on nonmyeloid neoplasm samples, which included CD9, CD10, CD19, CD20, CD22, CD24, CD34, CD38, CD58, and CD45 (Table S1). The FMO set up, where anti-CD9 FITC was solely omitted in the same antibody panel, was performed in parallel in 13 normal/nonmyeloid neoplasm samples.

Data acquisition was performed on a Navios EX flow cytometer and analyzed with Kaluza software (Beckman Coulter). The instrument was verified daily for optical alignment, fluidic stability, and optical sensitivity using Flow-Check Pro and Flow-Set Pro Fluorospheres (Beckman Coulter).

### Gating strategy and analysis

After doublet and debris exclusion, gating based on CD45 vs side scatter was used to separate populations into lymphocytes (high CD45, low side scatter), monocytes (high CD45, intermediate side scatter), granulocytes (variable CD45 and high side scatter), and blasts (dim CD45 and low side scatter).

The AML cases were confirmed by evaluating the MFC immunophenotype, morphologic, cytogenetic, and molecular data. The abnormal CD34-positive myeloid blasts were differentiated from immature B cells in the Pre-B tube by assessing CD10, CD19, CD22, and CD24. Isolated weak/partial CD19 expression was not sufficient to alter myeloid blast lineage assignment in our analysis. Myeloid blasts were all negative for CD10, CD22, and CD24. If the myeloid blast population was CD34 negative or subset positive, a similar approach excluding immature B cells to identify the dim CD45 myeloid blast population in the blast gate was used.

Gating of normal CD34-positive myeloid blasts in the Pre-B tube used a similar approach as noted previously for leukemic blasts. The HSC subset was further isolated by gating on the dim to absent CD38 component. This subset was slightly brighter for CD34 and CD45 compared to the CD38 uniformly positive CMP component. Normal granulocytic subsets were isolated first by selecting the granulocyte gate, then further by CD24 vs CD10, with promyelocytes being CD24 negative and CD10 negative, myelocytes being CD24 positive and CD10 negative, and neutrophils being CD10 and CD24 positive (Figure S1).^29^ Given that eosinophils express bright CD9^2^ and are coincident with myelocytes (CD24 positive and CD10 negative), myelocytes were excluded from further analysis. Normal monocytes were isolated first by selecting the monocyte gate and further refined as needed to exclude events from the granulocytes or lymphocyte region (CD10, CD19, CD22, CD24 negative). Mature B cells were initially isolated by selecting the lymphocyte gate and then by selecting CD19 positive and CD20 positive events that were negative for CD10. Hematogones were initially isolated by selecting the blast gate and then selecting the CD10 positive- CD19 positive, and CD38 positive population. Hematogone stages were defined by gating on the slightly brighter CD10, slightly dimmer CD19, and dimmer CD45 population with variable CD34 expression in stage 1 and by gating on the slightly dimmer CD10, slightly brighter CD19-positive, and slightly brighter CD45-positive population with mostly absent CD34 expression in stage 2. The CD9 GMFI was calculated using Kaluza software for the following normal subsets (if the population was confidently isolatable, without significant nonspecific deposition): myeloid blasts (including the HSCs and CMPs in cases with >30% HSCs of the CD34+/CD19– progenitors), promyelocytes, neutrophils, monocytes, mature B cells, and hematogones (stages 1 and 2) as well as FMO controls.

### Statistics

Log-transformation (base e) was conducted for the comparisons of CD9 GMFI. The statistical comparisons for CD9 expression included within-group (eg, CD9 in bone marrow vs CD9 in peripheral blood for the same group of patients) and between-group comparisons (eg, CD9 across the groups of patients with different AML subtypes vs normal myeloid blasts). For within-group comparisons, paired *t* tests were conducted. For between-group comparisons, *t* tests were conducted for 2 independent groups, and 1-way analyses of variance were conducted for 2 or more independent groups. Post hoc pairwise comparisons with Tukey adjustment were performed when at least 2 independent groups were involved in comparisons. All statistical tests were 2-sided at a significance level of less than .05. All statistical analyses were performed using SAS 9.4 (SAS Institute).

## RESULTS

### Normal cell subsets

**Figure 1:**
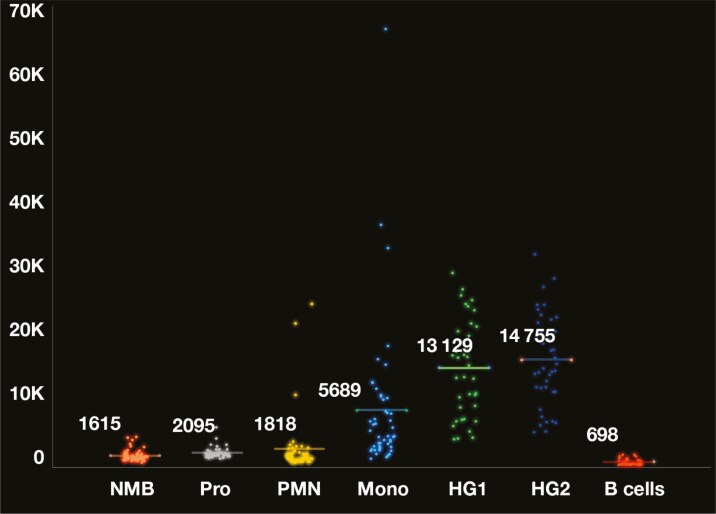
Comparison of CD9 geometric median fluorescence intensity (GMFI, logical scale) on normal cell subsets. Normal myeloid blast (NMB) GMFI range: 707-4662, promyelocyte (Pro) GMFI range: 1315-5481, neutrophil (PMN) GMFI range: 737-24 800, monocyte (Mono) GMFI range: 1357-66 487, stage 1 hematogone (HG1) GMFI range: 4245-29 439, stage 2 hematogone (HG2) GMFI range: 5287-32 269, normal mature B-cell (B-cell) GMFI range: 392-1921.

Of the 69 normal/nonmyeloid neoplasm cases, only normal myeloid blasts (among the populations of interest) were consistently evaluable in all cases (65 bone marrows and 4 peripheral bloods). Specific cell-type analysis was excluded if the cell type of interest numbers were too low or not confidently isolatable, or the cell type was abnormal (eg, an abnormal B-cell population). CD9 expression was lowest in normal mature B cells (n = 41; GMFI: 698), followed sequentially by normal myeloid blasts (n = 69; GMFI: 1615; Figure S2), neutrophils (n = 49; GMFI: 1818), and promyelocytes (n = 55; GMFI: 2095), with intermediate expression in monocytes (n = 48; GMFI: 5689), and highest in hematogones (stage 1 [n = 37; GMFI: 13 129] and 2 [n = 40; GMFI: 14 755]) **Figure 1**. Although there was no statistically significant difference in GMFI between neutrophils and promyelocytes (Tukey test *P* = .22), there was between neutrophils and monocytes (Tukey test *P* < .001), as well as between promyelocytes and monocytes (Tukey test *P* < .001). Even though the GMFI was overall low on granulocytes, they could demonstrate variable expression with a “bottom-heavy” appearance (showing a subset or a “tail” of expression), with most of the population being dim to negative (Figure S1).

**Figure 2:**
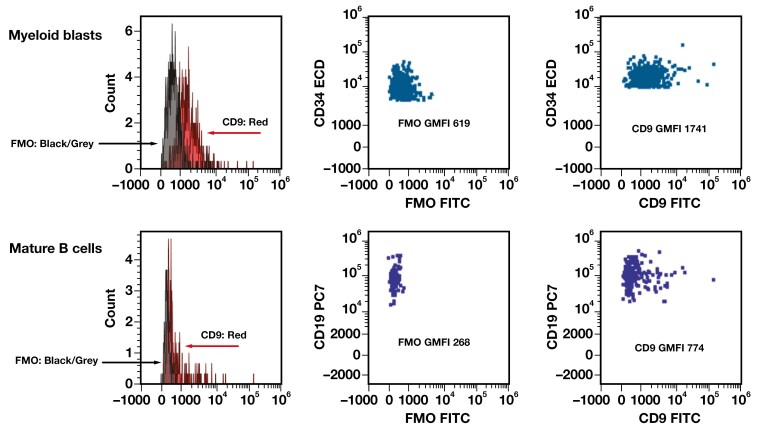
Representative histograms and dot plots of normal myeloid blasts and normal mature B cells with the fluorescence minus 1 (FMO) control. Top row, normal myeloid blasts; bottom row, normal mature B cells.

**Figure 3:**
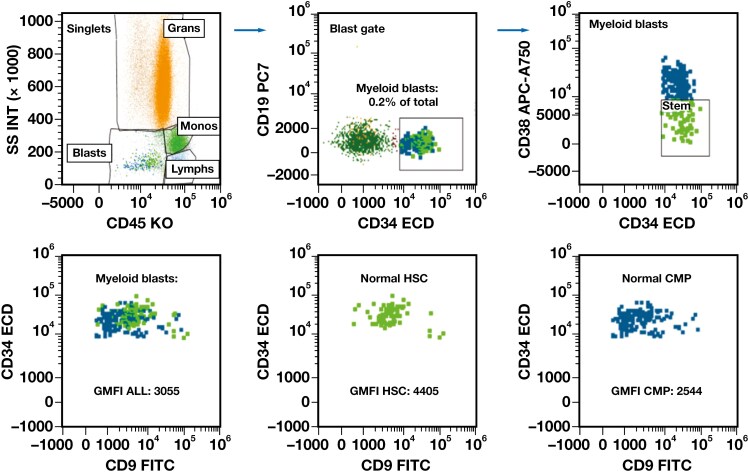
Example of normal myeloid blast expression from a patient with a nonmyeloid neoplasm (blood). The upper panel isolates the normal CD34-positive, CD19-negative myeloid blasts with further separation into the normal hematopoietic stem cells (HSCs, emphasized in bright green) and committed myeloid progenitors (CMPs, emphasized in blue) based on the CD34 and CD38 expression. Note the slightly higher expression of CD34 and CD45 of the HSCs compared to the CMPs. The lower panel demonstrates the normal CMPs (emphasized in blue) and normal HSCs (emphasized in bright green). Dot plots demonstrate the geometric median fluorescence intensity (GMFI) of the entire CD34-positive, CD19-negative myeloid blast population and the typically higher GMFI in the HSCs (“top heavy”) compared to the CMPs (“bottom heavy”).

Very low levels of CD9 expression were consistently observed in mature B cells and normal myeloblasts. The expression in these cell types was often more uniform; however, subset (or tail) expression was also occasionally observed, although not as significantly as in the granulocytes. When the GMFI was compared with the FMO paired samples, both mature B cells (n = 8; GMFI: 774 vs FMO GMFI: 268) and normal myeloid blasts (n = 13; GMFI: 1741 vs FMO GMFI: 619) showed statistically significantly higher CD9 GMFI (paired *t* test *P* < .0015 and *P* < .001, respectively; **Figure 2**). While there was a slight increase in the GMFI of normal myeloid blasts after administration of G-CSF (n = 8; GMFI: 1845) compared to no G-CSF (n = 61; GMFI: 1578), this increase was not statistically significant (*t* test *P* = .30). Assessment of CD9 GMFI in HSCs vs CMPs was performed in cases that had a robust HSC subset (n = 8), with higher GMFI in the HSCs compared to CMPs in all cases, which was statistically significant (paired *t* test *P* < .001) (**Figure 3**, Table S2). Please note that the case number was small in the FMO, G-CSF, and HSC vs CMP cohorts. We also further explored the clinical findings in 4 patients with a higher expression of CD9 on their normal myeloid blasts than the rest of the general cohort. We found that 3 of the 4 patients had concurrent viral infections (HIV, hepatitis B virus, and herpes simplex virus 1). The fourth patient did not have a reported infection and was undergoing therapy for plasma cell neoplasia. All patients additionally demonstrated variably elevated CD9 expression in the maturing myeloids, some significantly, while mature B cells remained low (Table S3).

### Acute myeloid leukemia

**Figure 4:**
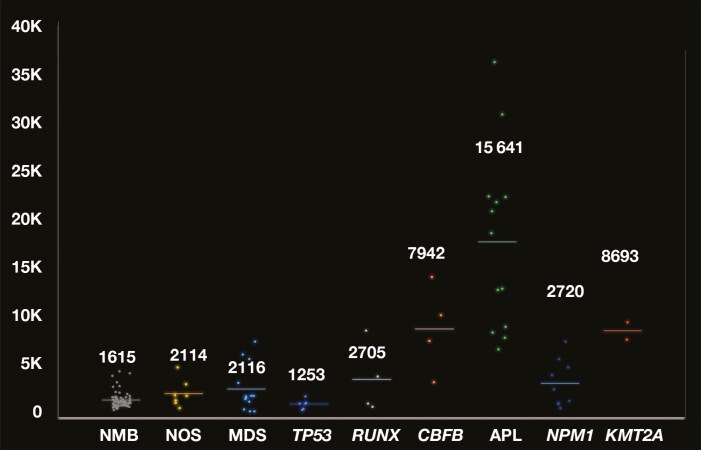
Comparison of CD9 geometric mean fluorescence intensity (GMFI, logical scale) of normal myeloid blasts (NBMs) and acute myeloid leukemia (AML) subtypes. NMB GMFI range: 707-4662; AML not otherwise specified (NOS), GMFI range: 912-5064; AML with myelodysplasia (MDS)–related mutation or genetic alterations, GMFI range: 580-7664; AML with *TP53* mutation (*TP53*), GMFI range: 760-2124; AML with *RUNX1::RUN1T1* fusion (*RUNX1*), GMFI range: 1084-8794; AML with *CBFB::MYH11* fusion (*CBFB*), GMFI range: 3520-14 178; acute promyelocytic leukemia (APL) GMFI range: 6856-35 903; AML with *NPM1* mutation (*NPM1*), GMFI range: 927-7661; AML with *KMT2A* rearrangement (*KMT2A*), GMFI range: 7852-9624.

**Figure 5:**
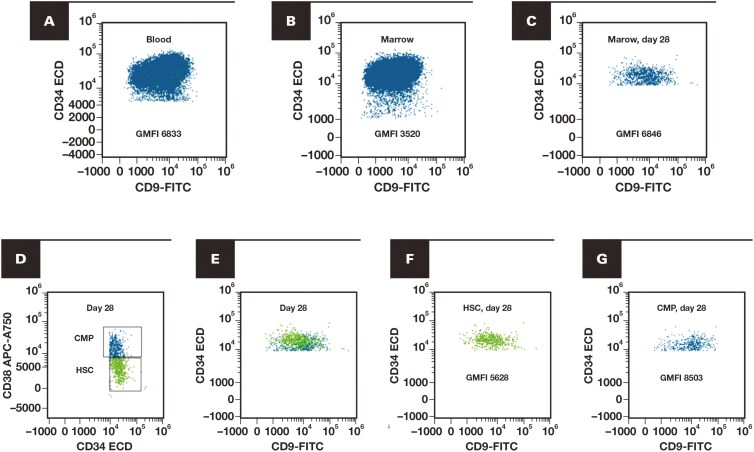
Dot plots of acute myeloid leukemia with *CBFB::MYH11* with paired peripheral blood and bone marrow samples, including evaluation of the hematopoietic stem cells (HSCs) and committed myeloid progenitors (CMPs). **A**, Leukemic blasts in the peripheral blood (PB) prior to treatment with geometric mean fluorescence intensity (GMFI, logical scale) of CD9. **B**, Leukemic blasts in the bone marrow (BM) prior to treatment with CD9 GMFI. **C**, Day 28 follow-up BM with 0.9% myeloid blasts with CD9 GMFI. **D**, Day 28 marrow separating HSCs (bright green) from CMPs (blue) based on CD34 vs CD38. **E**, **F**, The HSCs (bright green) exhibited abnormally lower CD9 GMFI than **G**, the CMPs (blue).

The 58 AML cases at presentation were sequentially collected: 6 *TP53* mutated; 4 *CBFB::MYH11*; 4 *RUNX1::RUNX1T1*; 13 myelodysplasia (MDS) related (rel), encompassing molecular and genetic subtypes; 13 *PML::RARA* (APL); 9 *NPM1* mutated; 2 *KMT2A* rearranged (*KMT2A*r); and 7 AML, not otherwise specified (NOS). Except for *TP53*-mutated AMLs, leukemic blasts demonstrated a tendency for a higher CD9 GMFI as compared to normal myeloid blasts with APL, AML with *CBFB::MYH11*, and AML with *KMT2Ar* expressing the highest levels (**Figure 4**, Figures S2 and S3). Some of the AML subtypes showed wide variability in the level of expression, with single cases demonstrating overlapping GMFI with other AML subtypes, as well as normal myeloid blasts. Given that the highest GMFI in normal myeloid blasts was 4662 (n = 69), a GMFI greater than 6000 seemed to be a likely indicator of abnormality **Figure 4**. Using 6000 GMFI as a cutoff, 22 cases of AML (38%) demonstrated a higher GMFI, although only 9 were not APL (20%). Additionally, entire population shifts or “top-heavy” patterns opposed to subset or uniform dim expression were additional potential indicators of abnormality. Although statistically significant differences were seen between some of the AML subtypes as well as normal myeloid blasts, only APL and MDS rel contained more than 10 cases in the AML cohorts **Table 1**. APL was also compared to normal promyelocytes, with CD9 expression significantly elevated in APL (Figure S4; Tukey test *P* < .001). Paired bone marrow and blood samples were studied in 10 cases, which showed differences in CD9 expression (some marked), although the finding was not statistically significant, again noting the small cohort (paired *t* test *P = *.3) (**Supplementary Table S4**, **Figure 5**).

**Table 1:** Comparison of Geometric Mean Fluorescence Intensity (GMFI) *P* Values Across Acute Myeloid Leukemia (AML) Subtypes and With Normal Myeloid Blasts

Characteristic	*TP53* mut (n = 6)	*CBFB::MYH11* (n = 4)	*RUNX1::RUNX1T1* (n = 4)	NOS (n = 7)	MDS rel (n = 13)	APL (n = 13)	*NPM1* mut (n = 9)	*KMT2A* rea (n = 2)
NMB	—^a^	<.001	.62	.93	.74^b^	<.001^b^	.13	<.001
*CBFB::MYH11*	<.001							
*RUNX1::RUNX1T1*	.39	.11						
NOS	.71	.004	>.99					
MDS rel	.55	.001	>.99	>.99				
APL	<.001	.4	<.001	<.001	**<.001^b^**			
*NPM1* mut	.14	.03	>.99	.99	**.98^b^**	**<.001^b^**		
*KMT2A* rea	<.001	>.99	.23	.03	**.019^b^**	.**88^b^**	.13	

^a^ *TP53* category showed lower GMFI than NMB, and therefore its *P* values are omitted here. ^b^Only APL and AML with MDS-related molecular or genetic changes had ≥10 cases in the cohort (bolded). Abbreviations: APL, acute promyelocytic leukemia; MDS rel, AML with myelodysplasia related mutations or genetic changes; mut, mutated; NMB, normal myeloid blasts; NOS, AML not otherwise specified; rea, rearranged.

Considering CD9 in follow-up cases, only 1 sample met all requirements noted previously, with that sample being AML with *CBFB::MYH11*. In this sample, blasts were not increased (0.9% of total), and MRD testing was negative (which does not include CD9 evaluation); however, the routine MFC panel, including CD9, demonstrated it was the only aberrancy (note abnormally high CMPs vs HSCs), with fluorescence in situ hybridization studies finding *CBFB* rearrangement in 2.5% **Figure 5**.

## DISCUSSION

Our study evaluated CD9 expression on leukemic myeloid blasts and across normal cell subtypes, including mature B cells, neutrophils, promyelocytes, hematogones (stages 1 and 2), monocytes, and normal myeloid blasts. This was a retrospective study evaluating a panel performed for clinical purposes (implemented in October 2022); therefore, there are several limitations, with one of the biggest limitations being that myeloid antigens were not in the same tube as the CD9. However, we were able to isolate the cell types based on CD45 vs side scatter and additionally utilizing CD10, CD19, CD22, CD24, and CD34, as described in the Methods section. Additional limitations include an overall low number of AML cases (with some subtypes containing <10 cases), all AML subtypes are not represented, the subsequent follow-up time is short (limiting the number of posttherapy evaluations), and non-AML therapies that the patients were receiving at the time of diagnosis were not assessed. Additionally, only 100 000 events were acquired in the panel, including CD9, limiting the sensitivity of that assay for MRD assessment. Although we performed statistical analysis and statistically significant differences were found, the reader should keep in mind the low number of cases in some of the cohorts.

Our findings are consistent with previous studies demonstrating granulocytes and mature B cells with the lowest GMFI of CD9, with monocytes and hematogones exhibiting higher levels of CD9 expression.^2,9^ Additionally, we further assessed normal myeloid blasts and mature B cells (marrow and blood) with FMO controls, showing that these populations are typically dim and very dim for CD9, respectively. Although FMO control for the myeloid blast itself would be ideal, given this information, we felt that normal mature B cells (and potentially neutrophils or promyelocytes) would be an adequate negative internal control in clinical practice for most cases (noting that the mature B-cell population cannot be neoplastic to function as a negative internal control).

In addition to demonstrating that normal myeloid blasts typically have lower CD9 expression compared to most leukemic blasts (with the exception of *TP53*-mutated AML, although that cohort included only 6 cases), we further subcategorized AMLs and analyzed CD9 expression across various AML subtypes. While statistically significant differences were identified, only APL and AML MDS rel contained at least 10 cases. Some of the AML subtypes demonstrated single-case outliers with a much higher expression than the rest of the cohort. Although we do not have a good explanation for that finding, if the cohorts contained higher numbers, perhaps the variance would be minimized. Our study confirms others’ findings that increased CD9 expression can help differentiate APL from most other AMLs,^10-12,14^ and leukemic blasts often have higher CD9 expression than normal myeloid blasts.^9^ Although there are certainly higher-sensitivity molecular methods to assess for APL posttherapy, we also determined that normal promyelocytes express much lower CD9 compared to leukemic APL blast equivalents (which was statistically significant), supporting Wen et al^14^ in the use of CD9 posttherapy. Potentially contradictory to our finding, others report elevated CD9 expression in AMLs with *NPM1* mutation, as compared to AML cases that were *NPM1* wild type.^9,15^ Although we did find that CD9 expression was overall higher in AML with the *NMP1* mutation compared to some AML subtypes and normal myeloid blasts, it was not across all AML subtypes (**Figure 4**, **Table 1**). Possible explanations for the findings could be the AML cohort, cohort size, fluorochrome choice, clone choice, and the use of the GMFI threshold for CD9 expression in our study (as opposed to positive vs negative). Likely the most significant difference is the cohort choice, given that we included APL and the other studies did not. Additionally, our study had far fewer numbers and a different method of assessment. Liu et al^15^ compared *NMP1*-positive cases (n = 160) to *NPM1*-negative AML (n = 178) using the French-American-British (FAB) classification; however, they did not include APL in their cohort. Additionally, they used more than 20% expression to consider a population positive (negative threshold was not explicitly described), although similar to our study, they used CD9 FITC (isotype IgG1, clone ALB6). In the *NPM1*-positive cohort, Liu et al^15^ also showed a higher number of CD9-positive cases in the monocytic (M4/5) as opposed to myeloid (M1/2) subtypes (*P *< .001); however, we did not have enough cases in our cohort to further confirm that finding.^15^ They additionally showed that monocytic NPM1-positive cases exhibited a higher positive rate for CD9 than monocytic NMP1-negative cases (*P *= .04). Touzet et al^9^ evaluated 112 patients with AML using the FAB criteria but excluded APL cases in their study. They included *FLT-3* and *NPM1* mutation analysis and separated AML cases as either positive or negative. They did not see an association with FAB subtype; however, they did report that CD9-positive AML cases had a tendency to be *NMP1* positive (*P* = .09). They used more than 20% expression to consider the myeloid blasts positive and also determined that granulocytes were negative for CD9, which may have been how they set their threshold for positivity. Similar to our study, they also compared median fluorescence intensity between normal and leukemic blasts, finding significantly higher expression on leukemic blasts. They used a brighter fluorochrome and a different clone than we used (anti-CD9-PE, clone H19a; BioLegend).^9^

We also evaluated normal HSCs, comparing them to CMPs, and our findings confirm that reported by Clay et al,^23^ with the HSC component having a higher expression of CD9 than the CMP component^23^; however, our numbers are small. Our findings on normal HSCs differ from what was reported by others, which may be secondary to the definition of CD9 expression, comparing normal HSCs to leukemic HSCs as opposed to normal myeloid blasts^9^ or grouping committed progenitors together without removing B lineage progenitors.^26^ Given the competing findings, further studies exploring the HSC component and its relation to the CMP component should be performed to further assess and compare if CD9 levels are consistently different. CD9 has also been reported as a potential marker of MRD testing with increased expression in both leukemic stem cells and leukemic blasts,^9,26^ which remains stable after therapy.^24^ Coustan-Smith et al^24^ found that CD9 was significantly overexpressed in AML compared to normal (*P* < .001). It was aberrantly expressed in both the CD38-positive and CD38-dim leukemic blasts and continued to be aberrant after therapy, making it a potentially useful MRD marker.^24^ Although we were interested to see if our findings would support the prior data, we had too few cases for confirmation.

In some instances, we found that caution should be exercised with the interpretation of CD9. In our cohort, among the 4 patients exhibiting the highest GMFI in normal myeloid blasts, 2 had viral infections, although the fourth was not exhibiting an apparent infectious or inflammatory process (potentially indicating that expression level could be influenced by medication). All patients showed at least 1 additional myeloid component that also expressed increased CD9. Therefore, in cases with elevated CD9 expression on myeloid blasts, evaluation of CD9 on the other myeloid components may help determine if CD9 upregulation is universal and should be interpreted with caution. We additionally evaluated a small subset of patients receiving G-CSF, which caused a slight upregulation of CD9; however, the change was not statistically significant (the cohort is small). Given that we also found that CD9 expression is higher on the HSCs than on the CMPs (although the cohort is small), one might keep in mind that in cases with a prominent, normal HSC subset, higher levels of CD9 may be present with a more uniform CD9 expression appearance.^23^ Alternatively, when HSCs are present in normal/low numbers, the CD34-positive, CD19-negative progenitors demonstrate a “bottom-heavy” pattern, given that CMPs are typically present in higher numbers than HSCs. Based on our data, a GMFI greater than 6000 (the highest GMFI in normal myeloid blasts was 4662) could be used as an indicator of abnormality **Figure 4**; however, additional studies will be necessary to confirm the spectrum of expression. Further studies exploring CD9 on the HSC subset, as well as the effects of G-CSF, infectious or inflammatory processes, and other potential confounding factors (such as medications), will be necessary for more confident application of these findings.

## CONCLUSION

The primary focus of this study was to determine the magnitude of difference in CD9 expression between leukemic blasts and normal blasts, determine the best internal control, and decide if it was an antigen worth pursuing for incorporation as an MRD marker. The focus was not to use it for AML classification, which others have studied with much larger cohorts; however, we did appreciate the difference between subtypes. Although some of the differences were statistically significant, the findings should be considered in the context of the small numbers of the cohorts. Leukemic blasts often have higher expression levels of CD9 than normal myeloid blasts, which are dim to negative for CD9 in most cases. In many cases, the incorporation of GMFI was additive, gaining confidence in the magnitude of the expression shift (or lack thereof). Given that with normal myeloid blasts, there could be a “tail” or subset expression, we considered whole population shifts and lower levels of CD9 expression in the CD38-negative HSC subset compared to the CD38-positive CMP subset more helpful in defining abnormality, rather than using subset expression of the entire population. Mature, normal B cells and myeloid blasts had the lowest level of expression, followed by neutrophils and promyelocytes, with mature B cells being a better internal control, given there was less of a tendency for upregulation of CD9 in different settings than granulocytes. CD9 is variably expressed in AML, with APL, AML with *CBFB::MYH11*, and AML with *KMT2A*r having the highest levels, although it should be noted that the cohorts are small. High levels of CD9 can also help distinguish APL from *NPM1-*mutated AML in most cases, although further studies with larger numbers will be necessary for confirmation. We also found that normal promyelocytes express lower levels of CD9 than leukemic blasts of APL, supporting the findings of Wen et al^14^ that it can be used as another marker to help define abnormality in MRD testing.^14^ Normal HSCs express slightly higher levels of CD9 than CMPs, with increased and/or atypical expression patterns potentially being useful to define abnormality in AML posttherapy, although further studies to confirm these findings will be necessary. In clinical practice, we have utilized expression of CD9 (in conjunction with other markers) primarily in follow-up AML samples to assist in determining whether the myeloid blast population is abnormal, looking for whole population shifts and comparing the HSCs to the CMPs. We are also further exploring its potential use as an AML MRD marker and hoping this study will create interest from others to additionally evaluate if CD9 will be a robust marker for AML MRD assessment in non-APL subtypes.

## Supplementary material

Supplementary material is available at *American Journal of Clinical Pathology* online.

aqaf087_suppl_Supplementary_Tables_S1-S4_Figures_S1-S4
