# Supplementary material for: CD9: Differential expression of normal bone marrow cellular components and leukemic myeloid blasts
Source: Am J Clin Pathol. 2025 Sep 17;164(5):694–703. doi: 10.1093/ajcp/aqaf087 (PMC12629872; doi:10.1093/ajcp/aqaf087)
Supplement: aqaf087_suppl_Supplementary_Tables_S1-S4_Figures_S1-S4 [file aqaf087_suppl_supplementary_tables_s1-s4_figures_s1-s4.pdf]

Supplementary Table S1. Acute leukemia flow cytometry panel for first diagnostic time point

Tube name to include Antibody, Fluorochrome and Clone		
T/NK TUBE<br>CD57 FITC (NC1)<br>TCR αβ PE (IP26A)<br>CD14 ECD (RMO52)<br>TCR γδ PC5.5 (IMMU510)<br>CD4 PC7 (SFCI1)<br>CD16 APC (3G8)<br>CD56 APC-A700 (N901 NKH-1)<br>CD3 APC-A750 (UCHT1)<br>CD8 PB (BP.11)<br>CD45 KO (J33)	PRE-B TUBE<br>CD9 FITC (ALB6)<br>CD24 PE (ALB9)<br>CD34 ECD (581)<br>CD10 PC5.5 (ALB1)<br>CD19 PC7 (J3-119)<br>CD58 APC (AICD58)<br>CD22 APC-A700 (SJ10.1H11)<br>CD38 APC-A750 (LS198.4.3)<br>CD20 PB (B9E9 HRC20)<br>CD45 KO (J33)	GRANS TUBE<br>CD64 FITC (22)<br>CD123 PE (9F5)<br>CD34 ECD (581)<br>CD56 PC5.5 (N901 NKH-1)<br>CD7 PC7 (8H8.1<br>CD13 APC (Immu103.44)<br>CD11c APC-A700 (BU15)<br>CD16 APC-A750 (3G8)<br>CD15 PB (80H5)<br>CD45 KO (J33)
T-CELL TUBE<br>CD4 FITC (13B8.2)<br>TCR αβ PE (IP26A)<br>CD14 ECD (RMO52)<br>TCR γδ PC5.5 (IMMU510)<br>CD7 PC7 (8H8.1)<br>CD5 APC (BL1a)<br>CD2 APC-A700 (39C1.5)<br>CD3 APC-A750 (UCHT1)<br>CD8 PB (BP.11)<br>CD45 KO (J33)	MYELOID TUBE<br>CD11b FITC (Bear1)<br>CD24 PE (ALB9)<br>CD34 ECD (581)<br>CD117 PC5.5 (104D2D1)<br>CD13 PC7 (366 My7)<br>CD33 APC (D3HL60.251)<br>CD10 APC-A700 (ALB1)<br>CD16 APC-A750 (3G8)<br>HLA-DR PB (Immu-357)<br>CD45 KO (J33)	TdT-MPO TUBE<br>cTdT FITC (HT-1+HT-4+HT-8+HT-9)<br>cMPO PE (CLB-MPO-1)<br>CD13 ECD (SJ1D1)<br>cCD79a PC5.5 (HM47)<br>CD7 PC7 (8H8.1)<br>CD33 APC (D3HL60.251)<br>cCD22 APC-A700 (SJ10.1H11)<br>CD34 APC-A750 (581)<br>cCD3 PE (UCHT1)<br>CD45 KO (J33)
CLL TUBE<br>FMC7 FITC (FMC7)<br>Lambda PE (PGH Lambda)<br>CD19 ECD (J3-119)<br>CD38 PC5.5 (LS198.4.3)<br>CD200 PC7 (OX-104)<br>Kappa APC (PGH Kappa)<br>CD23 APC-A700 (9P25)<br>CD5 APC-A750 (BL1a)<br>CD20 PB (B9E9 HRC20)<br>CD45 KO (J33)	MONO TUBE<br>CD64 FITC (22)<br>HLA-DR PE (Immu-357)<br>CD14 ECD (RMO52)<br>CD56 PC5.5 (N901 NKH-1)<br>CD4 PC7 (SFCI1<br>CD33 APC (D3HL60.251)<br>CD11c APC-A700 (BU15)<br>CD16 APC-A750 (3G8)<br>CD15 PB (80H5)<br>CD45 KO (J33)	

All 8 tubes are run in new cases of acute leukemia. All antibodies are from Beckman Coulter, with the clone in

Supp. Fig. 1. Gating strategy to define myeloid blasts, neutrophils, promyelocytes and monocytes

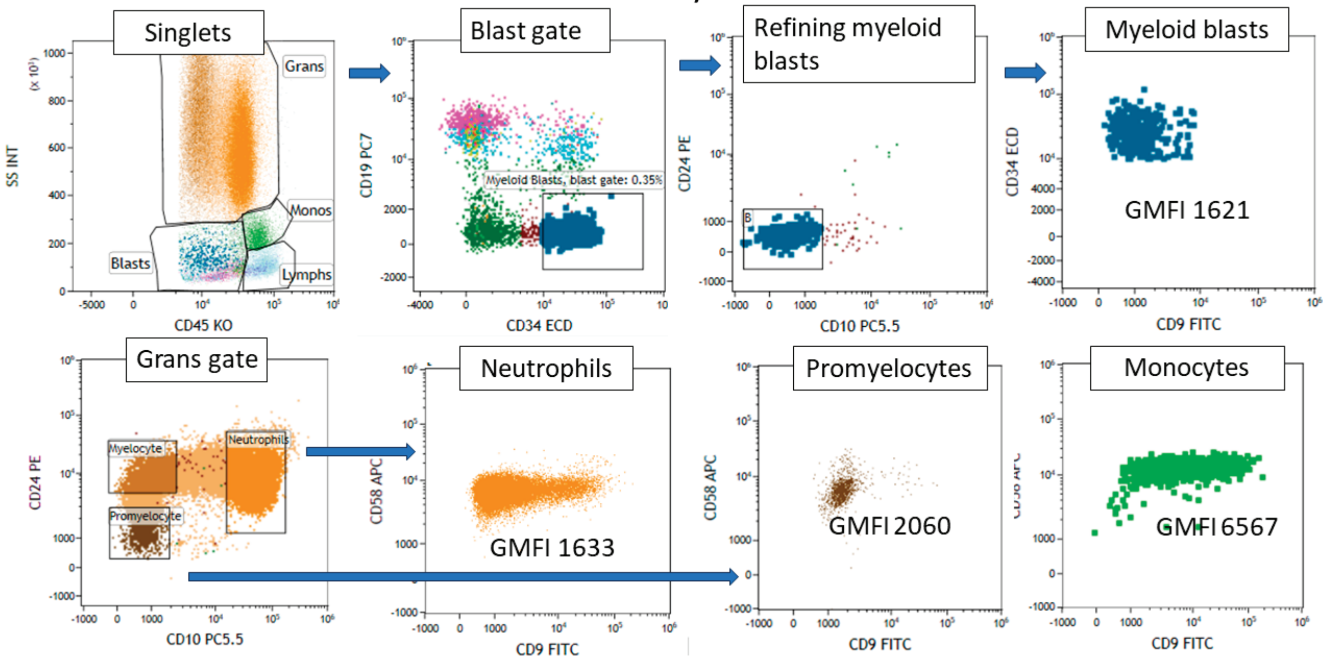

Supplementary Figure S1. Example of gating strategy to define myeloid blasts, neutrophils, promyelocytes and monocytes. Top row demonstrating CD45 vs side scatter gates with further myeloid blast isolation. Bottom row demonstrating isolation of neutrophils and promyelocytes using CD10 and CD24. Monocytes were refined using absence of CD10, CD22 and CD24. Note the neutrophils in the bottom row are mostly negative for CD9, with subset expression, giving a “bottom heavy” appearance. Grans = Granulocyte gate; Lymphs = Lymphocyte gate; Monos = Monocyte gate.

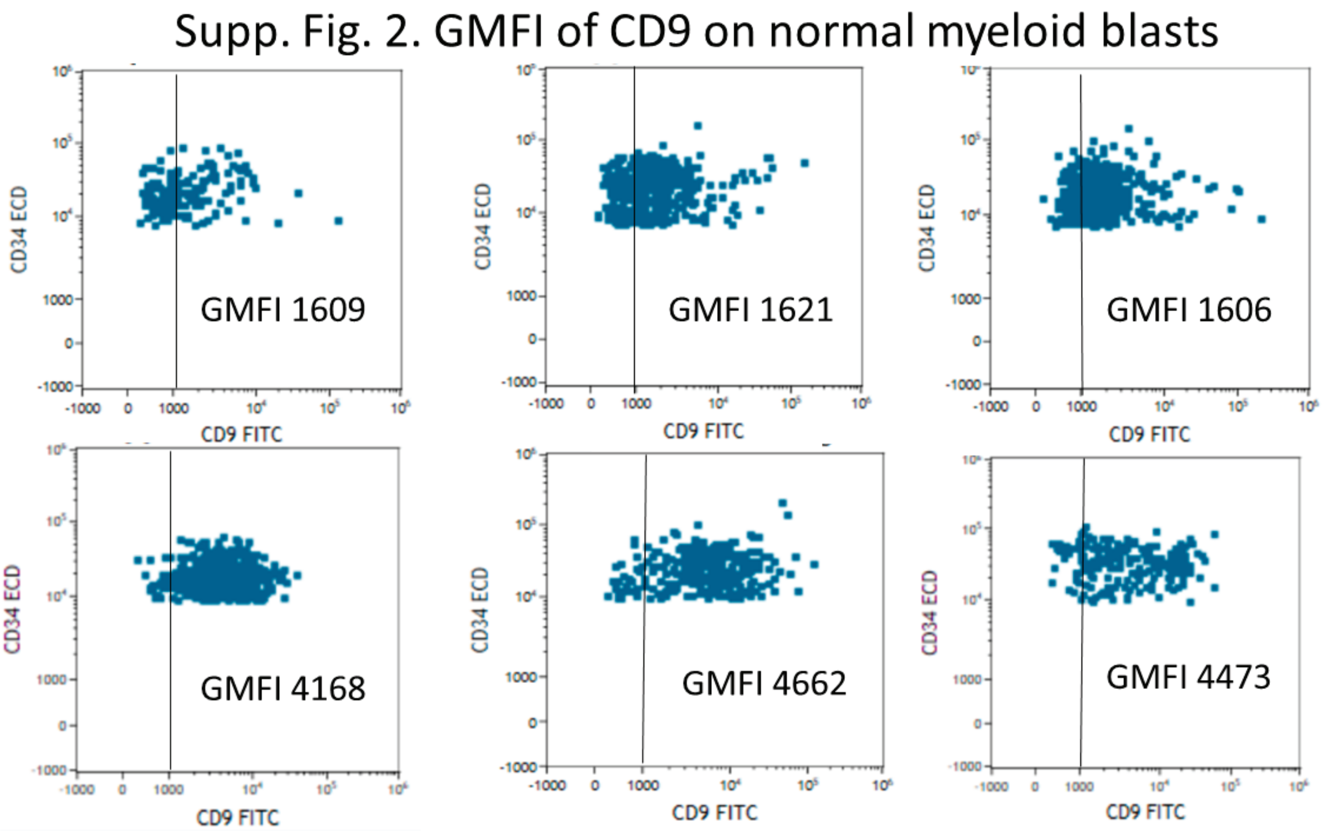

Supplementary Figure S2. Geometric mean fluorescence intensity of CD9 (GMFI, logical scale) of normal myeloid blasts ranged from 707 to 4662. Pictured in the top row are 2 myeloid blast dot plots close to the overall GMFI and the bottom row 2 myeloid blast dot plots on the highest end of the spectrum. Line at 1000 on all dot plots for visualization purposes only.

Supplementary Table S2. CD9 GMFI in normal CD34 positive, CD19 negative blasts separating into the hematopoietic stem cell (HSC) and committed myeloid progenitor (CMP).

Sample site	HSC	CMP
1. PB	3933	2386
2. BM	3616	1891
3. BM	4407	3709
4. PB	5032	4264
5. PB	3057	1713
6. BM	3061	2227
7. BM	2414	1182
8. PB	4509	3003

Abbreviations: BM, bone marrow; PB, peripheral blood.

Supplementary Table S3. Non-myeloid neoplasm patients (n = 4) with the highest CD9 GMFI on normal myeloid blasts and variably increased expression (some quite significant) on other myeloid elements.

Patient No.	Infectious Agent	Myeloid blast GMFI	Promyelocyte GMFI	Neutrophil GMFI	Monocyte GMFI	Mature B cell GMFI
1.	HIV	3550	3472	10 981	33 230	875
2.	HBV	4473	5481	24 800	66 487	491
3.	HSV1	4662	5753	21 799	36 844	391
4.	n/a	4168	4370	3373	15 537	n/a

Normal mature B cell expression remained low. Abbreviations: GMFI, geometric mean fluorescence intensity; HBV, hepatitis B virus; HIV, human immunodeficiency virus; HSV1, herpes simplex virus 1; n/a, not applicable.

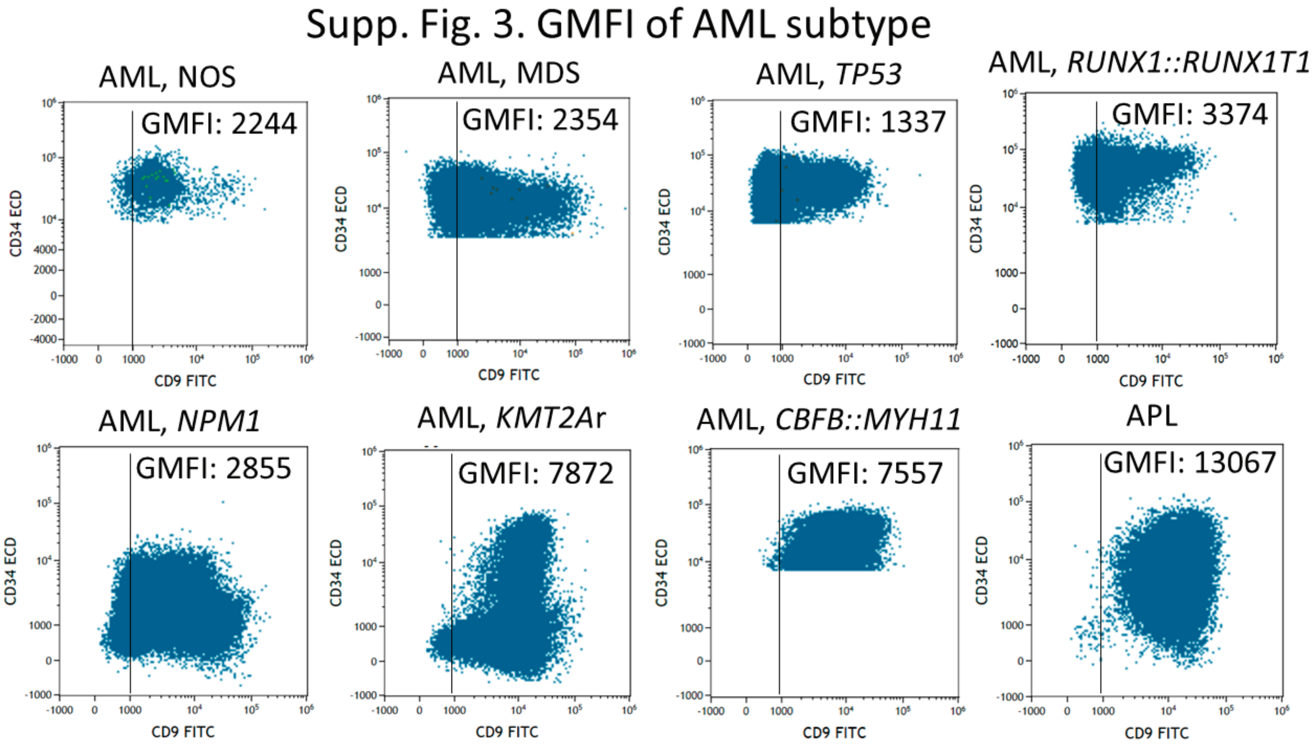

Supplementary Figure S3. Representative dot plot of each acute myeloid leukemia (AML) subtype that is close to the overall geometric mean fluorescence intensity (GMFI) for that subtype. Line at 1000 on all dot plots for visualization purposes only.

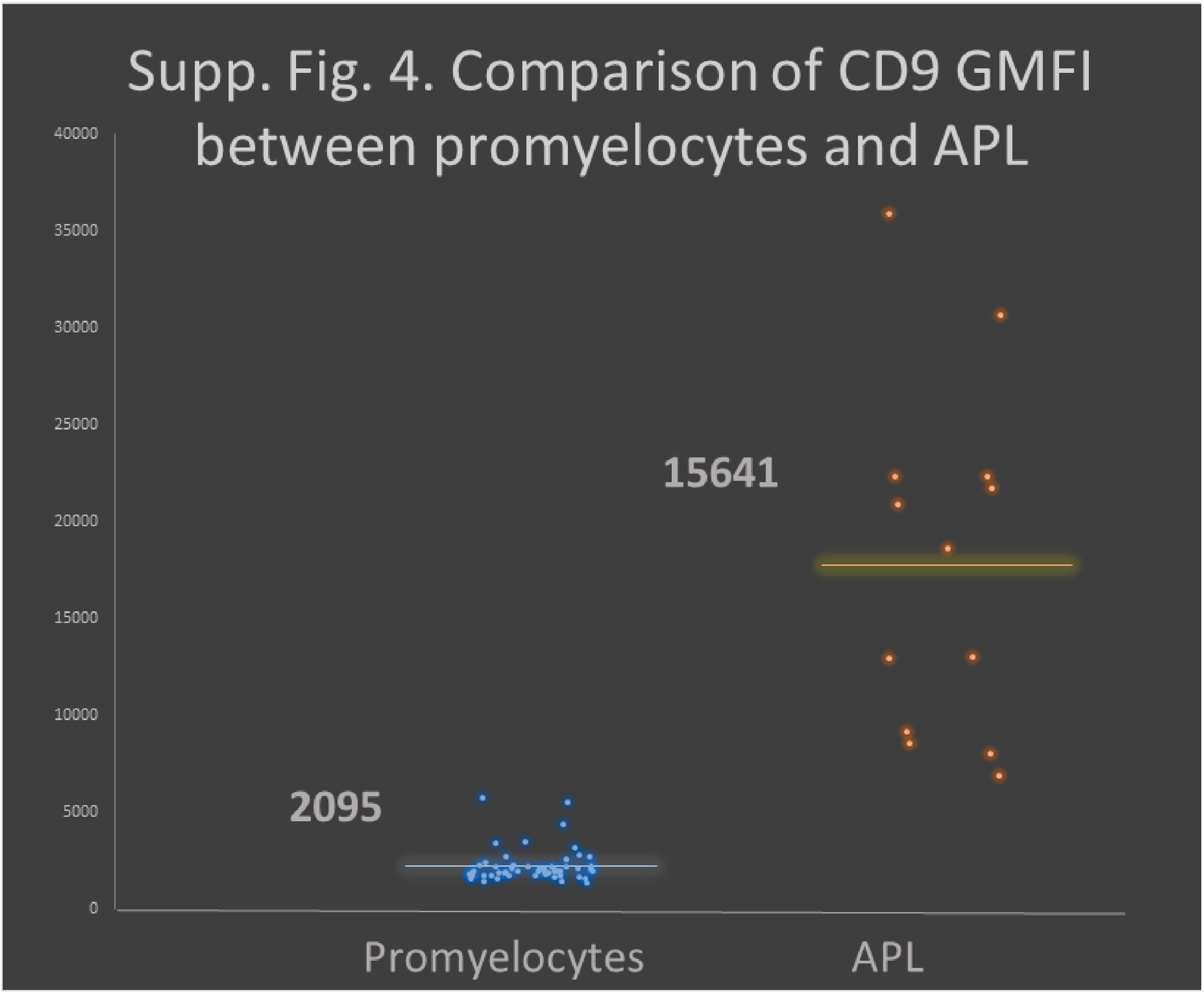

Supplementary Figure S4. Comparison of CD9 geometric mean fluorescence intensity (GMFI, logical scale) between normal promyelocytes and acute promyelocytic leukemia (APL). Promyelocytes: GMFI range: 1315-5481, APL: GMFI range 6856-35903.

Supplementary Table S4 Geometric mean fluorescence intensity (GMFI) of leukemic myeloid blasts in 10 paired blood and marrow samples.

**Sample No.**	**AML subtype**	**Bone marrow GMFI**	**Peripheral blood GMFI**
1	APL	30 636	15 567
2	APL	21 755	20 444
3	*NPM1* mut	1664	3755
4	*NPM1* mut	4257	3728
5	*NPM1* mut	5076	5543
6	*NPM1* mut	2857	5188
7	NOS	1713	3676
8	*TP53* mut	1400	1235
9	*CBFB::MYH11*	3520	6833
10	MDS rel	2244	1917

Abbreviations: AML, acute myeloid leukemia; APL, acute promyelocytic leukemia; MDS rel, myelodysplasia-related mutations or genetic changes; mut = mutation; NOS = not otherwise specified.
